# Prevalence and correlates of current suicidal ideation in women with premenstrual dysphoric disorder

**DOI:** 10.1186/s12905-022-01612-5

**Published:** 2022-02-11

**Authors:** Anna Wikman, Julia Sacher, Marie Bixo, Angelica L. Hirschberg, Helena Kopp Kallner, C. Neill Epperson, Erika Comasco, Inger Sundström Poromaa

**Affiliations:** 1 Department of Women’s and Children’s Health, Uppsala University Hospital, Uppsala University, 751 85 Uppsala, Sweden; 2 Emotion Neuroimaging (EGG) Lab, Max Planck Institute for Human Cognitive and Brain Sciences, Leipzig, Germany; 3 Department of Clinical Sciences, Umeå University, Umeå, Sweden; 4 Department of Women’s and Children’s Health, Karolinska Institutet, Stockholm, Sweden; 5 Department of Gynecology and Reproductive Medicine, Karolinska University Hospital, Stockholm, Sweden; 6 Department of Clinical Sciences at Danderyd Hospital, Karolinska Institutet, Stockholm, Sweden; 7 Department of Obstetrics and Gynecology, Danderyd Hospital, Stockholm, Sweden; 8 Department of Psychiatry, University of Colorado School of Medicine, Aurora, CO USA; 9 Department of Neuroscience, Science for Life Laboratory, Uppsala University, Uppsala, Sweden

**Keywords:** Premenstrual dysphoric disorder (PMDD), MADRS-S, Prevalence, Suicidal ideation, Women’s health

## Abstract

**Background:**

Although previous studies report an association between Premenstrual Dysphoric Disorder (PMDD) and suicidal ideation, most studies have only established a provisional and retrospective diagnosis of PMDD fundamentally invalidating the diagnosis. Therefore, the aim of this study was to describe the prevalence and to explore correlates of current suicidal ideation in the late luteal phase in women with prospectively assessed and confirmed PMDD.

**Methods:**

Participants were 110 women who attended the pre-randomization baseline visit of two randomized placebo-controlled clinical trials between January 15, 2017 and October 19, 2019. PMDD was diagnosed prospectively in line with DSM-5 criteria. Current suicidal ideation was measured by the MADRS-S in the late luteal phase. Descriptive statistics were presented and logistic regression analyses were carried out to explore the association between psychosocial and health characteristics and current suicidal ideation, presenting unadjusted odds ratios (OR) and 95% confidence intervals (CI).

**Results:**

Current suicidal ideation was reported by nearly 40% of women with confirmed PMDD (n = 43, 39.1%). Previous psychological treatment for PMDD and higher depressive symptoms in the late luteal phase were positively associated with current suicidal ideation (OR 5.63, 95% CI 1.07–29.49, and OR 1.17, 95% CI 1.10–1.25, respectively), whereas higher ratings of self-rated health were associated with lower odds ratios for current suicidal ideation (OR 0.98, 95% CI 0.96–0.99).

**Conclusions:**

A substantial proportion of women with confirmed PMDD report current suicidal ideation in the late luteal phase. Results point to a need for better awareness and screening of suicidal ideation in women with PMDD.

## Background

Premenstrual dysphoric disorder (PMDD) is a cyclic, sex hormone-based mood disorder, encompassing a constellation of debilitating physical, cognitive, and affective symptoms present during the luteal phase, abating with menstruation. Psychological symptoms of PMDD include depression, anxiety, irritability and emotional lability [7]. PMDD is a severe and disabling form of premenstrual syndrome (PMS) and causes clinically significant distress with substantial impact on women’s everyday lives and psychiatric co-morbidity is common [11]. As many as 2–5% of reproductive-aged women experience PMDD [7].

Of relevance for the impact of the PMDD symptoms on function, previous studies have found retrospectively reported symptoms of PMDD to be positively associated with suicidal ideation, plans, and attempts [1, 19]. A number of different mechanisms have been proposed to explain the link between PMDD and suicidality including perimenstrual changes and interactions in emotional, behavioural, and physiological factors in hormone-sensitive women [15]. These factors include, but are not limited to, changes in negative affect, emotion regulation dysfunction, disturbances in and negative appraisals of social and interpersonal interactions, sensitivity to expected rewards and reward magnitude, executive function deficits and diminished inhibitory control, and sleep disturbances [15].

One meta-analysis of correlational studies of the menstrual cycle and objective health outcomes, including largely naturally cycling women, points to an increased risk of suicide deaths and suicide attempts during the menstrual phase, but found no consistent evidence to suggest an association between the menstrual cycle and suicidal ideation [10]. Results from a register-based prospective cohort study in Sweden, including more than 1.5 million women, showed more than double the risk of suicidal behaviour in women with a clinical indication of PMS or PMDD compared with population controls. However, the validity of the diagnosis was unknown in this study [22].

A recent systematic review reporting on suicidality in women experiencing PMDD, including 10 papers from between 2002 and 2018 focusing on suicidal thoughts and/or attempts, pertaining to the current classification of PMDD, showed suicidal thoughts, ideation, plans and attempts were strongly associated with experiences of PMDD [14]. However, as noted by the authors, the PMDD diagnosis should be considered provisional for all studies included in the review as these were retrospectively assessed. Notably, in order to meet the Diagnostic and Statistical Manual of Mental Disorders, 5th edition (DSM-5) criteria for PMDD, prospective charting of two symptomatic cycles is required. A PMDD diagnosis based on retrospective self-report fundamentally invalidates the diagnosis, is prone to false positives and is likely only weakly associated with prospective PMDD diagnosis [4].

Therefore, the aim of this study was to describe the prevalence and to explore correlates of current suicidal ideation in the late luteal phase in women with prospectively assessed and confirmed PMDD.

## Methods

### Participants and procedure

Participants were women (females, identifying as women at enrollement) who attended the pre-randomization baseline visit of two investigator-initiated, multicenter, double-blind, randomized placebo-controlled clinical trials where participants were treated with either (1) ulipristal acetate 5 mg daily (Gedeon Richter) during three 28-day treatment cycles [3], or (2) SSRI Escitalopram 20 mg (Lundbeck) for two weeks during the luteal phase of one cycle. Eligibility criteria for both trials were: women 18–46 years of age, healthy, with regular menstrual cycles, who met diagnostic criteria for PMDD. The MINI International Neuropsychiatric Interview [20] was used to screen for and exclude contraindications for participating in the later trial including current psychiatric disorders, ongoing drug or alcohol abuse, previous hospitalisation for a psychiatric disorder or attempted suicide, and treatment with psychotropic medication or hormonal contraceptives during the previous three months.

The study was carried out at the Departments of Obstetrics and Gynecology at Uppsala University Hospital, the Karolinska University Hospital, Danderyd Hospital, and the University Hospital of Umeå between January 15, 2017 and October 19, 2019. Information about the study was distributed via advertisements in local newspapers and social media, but also offered to women attending any of the hospital clinics included during the recruitment period. Potential participants attended a screening visit to determine trial eligibility and written informed consent was obtained. Following two diagnostic cycles, eligible women attended the pre-randomization baseline visit, scheduled in the late luteal phase. The study was approved by the Regional Ethical Review Board (2016/184) and the Medical Products Agency in Sweden. The clinical trial identifiers are (1) EUDRA-CT 2016-001719-19 and (2) 2016-001217-25.

### Measures

Demographic, psychosocial and health characteristics included age, marital status, education, employment, parity, smoking, snus use (i.e., a moist oral tobacco product), prior use of hormonal contraceptives, previous drug treatment for PMDD, previous psychological treatment for PMDD and self-rated health, as measured by the EQ-5D visual analogue scale (VAS). Self-reported history of depressive and anxiety symptoms, and luteal phase depression scores, as measured by the Montgomery–Åsberg Depression Rating Scale self-rated version (MADRS-S, [21]), were collected. MADRS-S was presented both as total score and categorised as no depression (scores < 13), mild (13–19), moderate (20–34) or severe depression (> 34) [18]. Satisfactory validity, acceptability, reliability and sensitivity to change of the MADRS-S has been demonstrated [8]. Using MINI as gold standard and a cutoff level at 12, sensitivity of the MADRS-S was 87.4% and specificity 36.4% [12].

To confirm and diagnose PMDD, a smart-phone application was used to complete the Daily Record of Severity of Problems (DRSP), for at least two menstrual cycles. The DRSP comprises 21 items describing premenstrual symptoms and 3 items concerning the dysfunction in daily life caused by these symptoms including psychological and physical symptoms [5]. We defined PMDD as > 50% increase [6], in at least five of 11 symptoms between the follicular (day 6–12) and luteal phase (day − 7 to − 1), according to the DSM-5. Percent increase was calculated as (mean luteal phase scores − mean follicular phase scores / mean follicular phase scores) × 100. Among the five symptoms, at least one should be a core PMDD symptom [7]. Diagnostic symptoms were at least moderate in severity (mean luteal phase score > 3.0), and disappeared during the follicular phase (mean follicular phase score < 2.0) [5]. Psychometric evaluation of the measure shows the DRSP to be a sensitive, reliable and valid measure of the symptoms and impairment criteria for PMDD [5].

Suicidal ideation *during the past three days* was assessed using the MADRS-S, developed to assess severity of depressive episodes [21]. The MADRS-S includes one item assessing suicide risk, which is rated from 0 (“Enjoys life or takes it as it comes”), 1 (no description), 2 (“Weary of life. Only fleeting suicidal thoughts”), 3 (no description), 4 (“Probably better off dead, suicidal thoughts are common, and suicide is considered as a possible solution, but without specific plans or intention”), 5 (no description) to 6 (“Explicit plans for suicide when there is an opportunity. Active preparations for suicide”). A score of 2 or above was considered ‘any’ current suicidal ideation, whereas a score of 4 or above was considered ‘acute’ current suicidal ideation. Women with current suicidal ideation were followed through the study, via the daily entries on the smartphone application, to ensure that depressive symptoms decreased in the follicular phase. Four women in the later trial (three on placebo) discontinued due to worsening mood symptoms, one of whom was referred to psychiatric specialist care.

### Statistical analyses

Data were presented using descriptive statistics describing means and standard deviations (SD) for numerical variables and numbers (n) and percentages (%) for categorical variables. To examine associations between demographic, psychosocial and health characteristics among women with confirmed PMDD and any current suicidal ideation, unadjusted logistic regression analyses were carried out using no suicidal ideation as reference. Results are presented as unadjusted odds ratios (OR) with their corresponding 95% confidence intervals (CI). Differences in symptoms on the DRSP among women with current suicidal ideation or no suicidal ideation were explored using independent samples t-test. All statistical analyses were performed using IBM SPSS Statistics version 27.0 (IBM Corp., Armonk, N.Y., USA).

## Results

Among 182 women assessed for eligibility 180 were screened, of whom 30 did not fulfill inclusion critera, 2 were pregnant, 27 withdrew consent and 11 were excluded for other reasons (e.g., lost contact, stopped scoring the DRSP diaries). Thus, 110 women fulfilled PMDD criteria and completed the MADRS-S at the pre-randomization baseline visit. Demographic, psychosocial and health characteristics are shown in Table 1. Women were aged 35.5 (SD = 6.0) years on average, most were married or cohabiting (n = 72, 65.5%), had a university degree (n = 83, 75.5%) and were working (n = 94, 85.5%). A total of 83 (75.5%) women reported previous drug treatment for PMDD, and some women (n = 8, 7.3%) reported previous psychological treatment for PMDD. One-third of women self-reported a history of depression (n = 35, 31.8%), and about 1 in 10 reported a history of anxiety (n = 13, 11.8%). MADRS-S scores revealed that 18.2%, 41.8% and 18.2% of participants experienced mild, moderate, and severe luteal phase depressive symptoms, respectively.

**Table 1:** Demographic, psychosocial and health characteristics among women with PMDD reporting any suicidal ideation compared with none

	Total sample n = 110 (100%)	Current suicidal ideation		
		None n = 67 (60.9%)	Any* n = 43 (39.1%)	OR (95% CI)
*Characteristics, n (%)*				
Age, mean [SD]	35.5 [6.0]	36.0 [5.7]	34.6 [6.5]	**–**
Age				
20–29	22 (20.0)	11 (16.4)	11 (25.6)	1
30–39	54 (49.1)	33 (49.3)	21 (48.8)	0.64 (0.23–1.73)
≥ 40	34 (30.9)	23 (34.3)	11 (25.6)	0.48 (0.16–1.44)
Marital status				
Partnered^a^	88 (80.0)	51 (76.1)	37 (86.0)	1
Single	22 (20.0)	16 (23.9)	6 (14.0)	0.52 (0.19–1.45)
Education				
University degree	83 (75.5)	47 (70.1)	36 (83.7)	1
Less than university degree	27 (24.5)	20 (29.9)	7 (16.3)	2.19 (0.84–5.74)
Employment				
Working	94 (85.5)	57 (85.1)	37 (86.0)	1
Studying	11 (10.0)	7 (10.4)	4 (9.3)	0.88 (0.24–3.22)
Other	5 (4.5)	3 (4.5)	2 (4.7)	1.03 (0.16–6.44)
Parity				
Nulliparous	45 (40.9)	23 (34.3)	22 (51.2)	1
Parous	65 (59.1)	44 (65.7)	21 (48.8)	0.50 (0.23–1.09)
Smoking				
No	101 (91.8)	63 (94.0)	38 (88.4)	1
Yes	9 (8.2)	4 (6.0)	5 (11.6)	2.07 (0.52–8.20)
Snus				
No	95 (86.4)	57 (85.1)	38 (88.4)	1
Yes	15 (13.6)	10 (14.9)	5 (11.6)	1.33 (0.42–4.21)
Previou hormonal contraceptives				
No	6 (5.5)	4 (6.0)	2 (4.7)	1
Yes	104 (94.5)	63 (94.0)	41 (95.3)	1.30 (0.23–7.43)
Previous drug treatment for PMDD				
No	27 (24.5)	17 (25.4)	10 (23.3)	1
Yes	83 (75.5)	50 (74.6)	33 (76.7)	1.12 (0.46–2.75)
Previous psychological treatment for PMDD^b^				
No	92 (83.6)	60 (96.8)	32 (84.2)	1
Yes	8 (7.3)	2 (3.2)	6 (15.8)	**5.63 (1.07–29.49)**
Self-rated health (EQ-5D VAS), mean [SD]	46.7 [23.6]	51.9 [23.2]	39.6 [22.6]	**0.98 (0.96–0.99)**
Self-reported history of depression^c^				
No	74 (67.3)	47 (71.2)	27 (62.8)	1
Yes	35 (31.8)	19 (28.8)	16 (37.2)	1.45 (0.65–3.32)
Self-reported history of anxiety				
No	97 (88.2)	60 (89.6)	37 (86.0)	1
Yes	13 (11.8)	7 (10.4)	6 (14.0)	1.39 (0.43–4.46)
Late luteal phase depression scores in the range of clinical depression**				
None	24 (21.8)	24 (35.8)	0 (0.0)	**–**
Mild	20 (18.2)	12 (17.9)	8 (18.6)	**–**
Moderate	46 (41.8)	29 (43.3)	17 (39.5)	**–**
Severe	20 (18.2)	2 (3.0)	18 (41.9)	**–**
Late luteal phase depressive symptom score**, mean [SD]	21.1 [10.1]	16.7 [9.0]	27.9 [7.9]	**1.17 (1.10–1.25)**

Significant odds ratios (OR) are marked in bold

^a^Partnered, including married, cohabitating, non-cohabitating partner

^b^Missing n = 10 (9.1%)

^c^Missing n = 1 (0.9%)

^*^cut-off ≥ 2 on item 9 MADRS-S “Weary of life. Only fleeting suicidal thoughts.”

^**^Assessed by MADRS-S

Current suicidal ideation was reported by nearly 40% (n = 43, 39.1%) of women in the study (Table 1), of whom eight (18.6%) reported acute suicidal ideation. Women who reported previous psychological treatment for PMDD were more likely to also report suicidal ideation during the late luteal phase (OR 5.63, 95% CI 1.07–29.49). Women with PMDD reporting current suicidal ideation also reported poorer self-rated health (mean 39.6, SD 22.6) than did women with PMDD and no suicidal ideation (mean 51.9, SD 23.2), where higher ratings of self-rated health were negatively associated with current suicidal ideation (OR 0.98, 95% CI 0.96–0.99). Those reporting current suicidal ideation also reported higher overall depressive symptom scores during the late luteal phase than women with no suicidal ideation (mean 27.9, SD 7.9 and mean 16.7, SD 9.0, respectively), where higher depressive symptoms were positively associated with current suicidal ideation (OR 1.17, 95% CI 1.10–1.25) among women with PMDD. No significant associations were observed for any of the remaining psychosocial and health characteristics included in the study. Women with PMDD reporting current suicidal ideation scored statistically significantly higher on the DRSP items feeling hopeless (mean 3.8, SD 0.8), feeling worthless or guilty (mean 3.8, SD 0.8), and feeling easily hurt (mean 4.0, SD 0.8) compared with women with PMDD and no current suicidal ideation (mean 3.3, SD 1.2; mean 3.3 SD 1.2; mean 3.5, SD 1.2; respectively).

## Discussion

In this study we found a notable proportion of women, nearly 4 in 10, with prospectively assessed and diagnosed PMDD, reported any current suicidal ideation during the late luteal phase. Comparatively, around 3% of the Swedish general population aged 16–84 years report some suicidal thoughts during a 12-month period, and the life-time prevalence of suicidal thoughts in Swedish women is 15% [17]. The prevalence of suicidal ideation observed in the present study appear somewhat higher than in previous reports [14]. It is important to acknowledge that differences in prevalence rates will depend greatly on varied criteria for defining suicidal ideation. Although both psychosocial and socioeconomic factors are associated with suicide [13], we found no associations between demographic characteristics and current suicidal ideation in this sample of women with PMDD. However, this is not surprising considering most women were highly educated, working and partnered. The observation that women with current suicidal ideation had previously been in contact with a psychologist for their PMDD and reported poorer self-rated health suggests that this population of women with PMDD have been and continue to be more severely ill. Although women with current psychiatric comorbidity or prior hospitalization for psychiatric disorders were excluded for the purposes of the trial from which the sample was included, women with a self-reported history of depression more frequently reported suicidal ideation. Notably, psychiatric comorbidity is not incompatible with the PMDD diagnosis if symptoms are unique in each disorder [9]. The exclusion of women with current psychiatric comorbidity may have led to an underestimation of the prevalence of suicidal ideation in women with PMDD in a real-world as opposed to a trial context. Women with PMDD and current suicidal ideation also reported greater hopelessness, worthlessness or guilt and more easily feeling hurt than did women with PMDD without suicidal ideation. Although the absolute difference in scores were small, yet statistically significant, women scoring higher on these items on the DRSP may warrant further attention also.

The major strength of this descriptive study is the use of real-time monitoring of symptoms through the menstrual cycle to confirm PMDD, greatly strengthening the validity of the diagnosis compared with previous studies. Due to the poor prospective validity of retrospectively reported premenstrual symptoms, a valid diagnosis requires evaluation of prospective daily symptom ratings as in the present study. Previous studies have largely included women with provisional and retrospectively assessed PMDD fundamentally invalidating the diagnosis, and may have included women with depressive episodes or other psychopathology instead [1, 16, 19]. Limitations include the somewhat limited sample size and the assessment of suicidal ideation. In the present study, one item from the MADRS-S [21] was used to identify women with any suicidal ideation. Although not gold-standard assessment of suicidal ideation, the suicide item on MADRS-S correlates strongly with the multi-item assessment scale, the Scale for Suicide Ideation [2], and is a commonly used screening instrument in Swedish health care settings. Defining suidical ideation as any suicidal thought, even fleeting thoughts might have overestimated the prevalence. However results remained unchanged when using acute suicidal ideation as outcome (data not shown). It is also worth noting that suicidal ideation, measured in the late luteal phase, might be more severe than other times under the menstrual cycle. Whether the risk of suicidal ideation or actual suicidal attempt varies under the menstrual cycle needs further studies.

## Conclusions

In conclusion, a substantial proportion of women with a prospective, gold-standard assessed, valid PMDD diagnosis report current suicidal ideation. Mirrioring the conclusions of a recent systematic review, women with PMDD should be considered a high-risk group for suicidality [14]. That is, women who are neurobiologically sensitive to hormone changes, as the case with PMDD, may be at increased risk of not only suicidal thoughts but also suicidal behaviour. The present results further point to a need for increased awareness among health care professionals and a need for improved and standardized screening of suicidal ideation in women with PMDD.

## Data Availability

Data are not publicly available due to ethical restrictions. Data that support the findings of this study are available from the corresponding author, upon reasonable request and with necessary ethics approval.

## Abbreviations

CI: Confidence interval
DRSP: Daily record of severity of problems
DSM-5: Diagnostic and statistical manual of mental disorders
MADRS-S: Montgomery–Åsberg depression rating scale self-rated version
OR: Odds ratio
PMDD: Premenstrual dysphoric disorder
SD: Standard deviation
VAS: Visual Analogue Scale
